# Transition of a Mallory-Weiss syndrome to a Boerhaave syndrome confirmed by anamnestic, necroscopic, and autopsy data: A case report

**DOI:** 10.1097/MD.0000000000013191

**Published:** 2018-12-10

**Authors:** Maria Cuccì, Fiorella Caputo, Giulio Fraternali Orcioni, Anna Roncallo, Francesco Ventura

**Affiliations:** a Department of Legal and Forensic Medicine, University of Genova, Genova; b Department of Clinical Pathology, S. Croce e Carle Hospital, Cuneo, Italy.

**Keywords:** Boerhaave syndrome, forensic autopsy, Mallory-Weiss syndrome, spontaneous esophageal perforation, sudden death

## Abstract

**Rationale:**

Spontaneous esophageal rupture (Boerhaave syndrome) is a rare, though frequently fatal, event. It is generally caused by a sudden increase in pressure inside the esophagus. In some cases, full-thickness perforations of the esophagus may develop from previous lesions that initially involve only the esophageal mucosa (Mallory-Weiss syndrome) and which, following further triggering events, give rise to a transmural lesion.

**Patient concerns:**

Here, we present the case of a 45-year-old subject who suddenly died of acute cardio-respiratory failure, an autopsy was performed to identify the cause of death.

**Diagnosis, interventions, and outcomes:**

The autopsy examination revealed a full-thickness rupture of the esophageal wall. Through the integration of necroscopy findings, anamnestic data, and histopathological examination, it has been possible to establish that complete esophageal rupture resulted from the evolution of a previous partial lesion of the esophageal wall, and that an untreated Mallory-Weiss syndrome evolved into a rapidly fatal Boerhaave syndrome.

**Lessons:**

This case shows that distal esophageal tears, rather than constituting a distinct entity, may be part of a spectrum of diseases and that a partial lesion of the esophageal wall caused by barogenic injury may evolve into a full-thickness rupture following further barotraumas.

## 1 Introduction

Esophageal perforations may be iatrogenic, traumatic, due to foreign bodies, related to other pathologies or spontaneous.^[1]^ Iatrogenic perforations are the most frequent (52.1% of cases), and include those caused by endoscopy or procedures carried out on the esophagus or nearby organs. Traumatic perforations result from penetrating wounds that tear the cervical and/or thoracic esophagus, and may be caused by road accidents.^[2]^ The ingestion of foreign bodies may cause erosion of the esophageal wall in areas of physiological narrowing, causing fistulization and sepsis.^[3]^ Disease-related perforations may occur in patients with esophageal diverticula, cancer, or infective esophagitis.^[4]^ Spontaneous lesions may involve only a part of the esophageal wall (Mallory-Weiss syndrome) or constitute a full-thickness rupture of the organ, giving rise to Boerhaave syndrome, which accounts for 8 to 56% of all esophageal perforations.^[1]^

Mallory-Weiss syndrome is characterized by longitudinal lacerations of the esophageal mucosa and is a common cause of gastrointestinal bleeding, accounting for 15% of hemorrhages of the upper digestive tract.^[5]^ The main symptoms may vary in severity according to the depth of the lesion, and are constituted by hematemesis (seen in about 40% of cases), melena (the onset symptom in about 30% of cases), hematochezia, abdominal pain, chest pain, tachycardia, syncope, hypotension, or hemorrhagic shock.

Boerhaave syndrome, which involves transmural esophageal rupture, is a clinical emergency, as it carries high mortality. The main symptoms include vomiting, chest pain (the chief symptom, present in 70% of patients), dyspnea, dysphagia, subcutaneous emphysema, tachycardia, fever, tachypnea, and epigastric pain. In rare cases, these esophageal perforations may be manifested by hematemesis or other signs of gastrointestinal bleeding, including melena.^[6]^ These symptoms, however, are not pathognomonic for Boerhaave syndrome, and may therefore be confused with the symptoms of other diseases, including pneumonia, acute myocardial infarction, pericarditis, and pancreatitis; the consequent, potential delay in reaching a diagnosis explains the high mortality rate of this pathology (between 10% and 25% in cases treated within 24 hours; up to 40–60% in those treated after 48 hours).^[7]^

Both syndromes seem to share the same pathogenic mechanisms; both are linked to increased pressure inside the esophagus, and therefore to a barotrauma, and occur more commonly in men with a history of alcohol abuse.^[8]^ These pathologies are usually associated to episodes of vomiting or retching after the consumption of alcoholic beverages or a heavy meal. Other precipitating factors include: severe coughing, acute asthma, epilepsy, constipation, childbirth, lifting weights, thoraco-abdominal traumas, and endoscopy maneuvers.^[9,10]^

Here, we describe the case of a 45-year-old man who died as a result of a spontaneous rupture of the esophagus. Integrated analysis of autopsy, anamnestic, and histopathological findings revealed that the fatal esophageal perforation was not manifested suddenly, but had developed from a previous partial wall lesion, which had been present for some days, thus indicating a transition from an initial Mallory-Weiss lesion to a Boerhaave syndrome.

## 2 Case report

**Figure 1:**
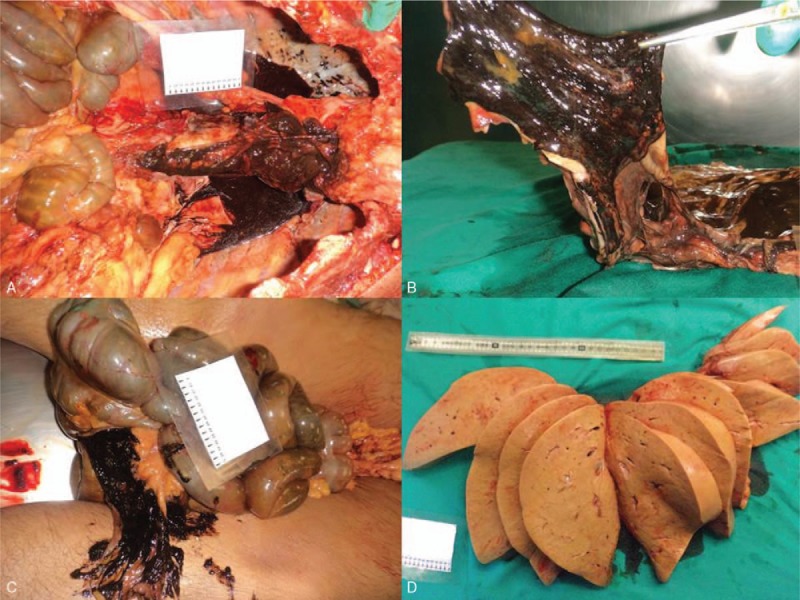
(A) Bilateral pleural effusion of brownish material. (B) Three centimeter esophageal tear in the right lateral wall of the inferior third. (C) Intestinal hemorrhage. (D) Marked hepatic steatosis.

**Figure 2:**
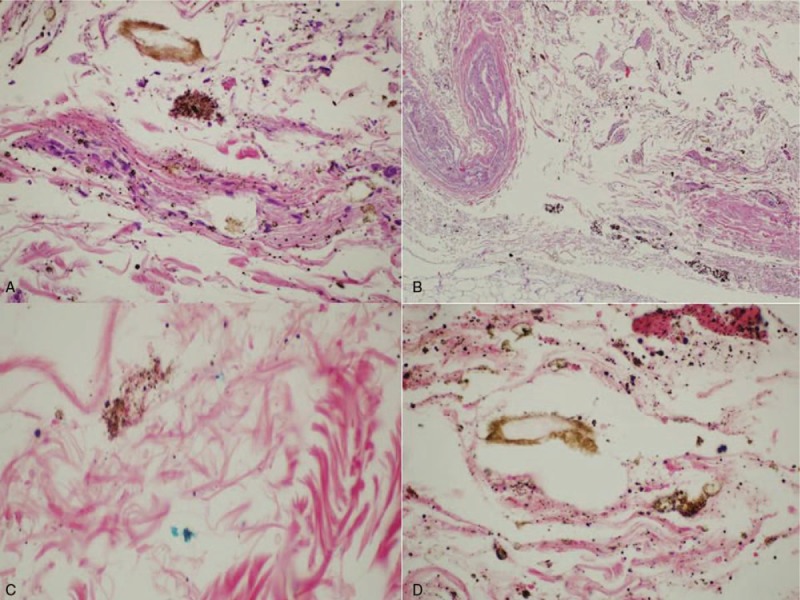
(A, B) Neutrophil infiltration of the esophageal mucosa and submucosa (H&E 40×), (H&E 4×). (C, D) Digested material and the presence of hemosiderin accumulations in the esophageal mucosa and submucosa (Perls 60×).

A 45-year-old man, who lived alone, alerted the emergency service and reported violent chest and abdominal pains and vomiting. On the arrival of the paramedics, about 20 minutes later, the man was found in a state of cardio-circulatory arrest and death was ascertained. The body was surrounded by copious traces of vomit and hematic material, which also soaked the man's clothing. From circumstantial data gathered during an interview with the victim's family members, it emerged that the man had been complaining of general malaise and thoraco-abdominal pain for about a week. Anamnesis revealed a history of alcoholism, arterial hypertension, and an ischemic stroke 5 years earlier. Autopsy was performed 2 days after the death. On external examination, the body presented coffee-colored material in the perioral region, on the hands and on the clothes; no traumatic lesions were observed. On internal examination, the heart weighed 305 g and presented slight myocardial sclerosis and patent, elastic coronary arteries. About 300 cm^3^ of coffee-colored material was found in the left pleural cavity, and 350 cm^3^ in the right cavity (Fig. 1A). Moreover, a hemorrhagic infiltration of the esophageal wall was noted. On the right lateral wall of the distal tract of the esophagus, about 4 cm from the stomach, a vertical transmural tear of 3 cm in length was observed (Fig. 1B). The jejunal loops were filled with digested blood and semisolid material (Fig. 1C); the liver weighed 2385 g and presented marked steatosis (Fig. 1D). Histopathlogical examination was subsequently carried out on organ samples taken during autopsy and stained with hematoxylin and eosin. Microscopic examination revealed the presence of a mainly granulocytic inflammatory infiltrate of the esophageal mucosa and submucosa (Fig. 2A, B), with collections of undigested alimentary material and accumulations of hemosiderin highlighted by means of Perl stain (Fig. 2C, D). Toxicological examinations were performed on blood and urine samples taken during autopsy, but did not reveal the presence of alcohol or substances of abuse. The findings that emerged from the autopsy and the subsequent histopathological analyses enabled the cause of death to be attributed to acute cardiorespiratory failure due to pleural effusion secondary to esophageal rupture.

## 3 Discussion

Esophageal pathologies and lesions are an infrequent cause of sudden death and are not commonly detected on autopsy. Deaths due to esophageal pathologies generally result from occlusion of the airways by tumors or foreign bodies, from intraluminal hemorrhages due to intravascular defects, or from perforations.^[11]^

Spontaneous esophageal rupture is defined as the complete destruction of the wall of the esophagus in the absence of pre-existing pathologies, and therefore in a previously healthy esophagus.^[12,13]^

Experimental data obtained on fresh cadavers with the medial portion of the esophagus clamped have revealed that a pressure of 150 mmHg is able to produce a tear at the level of the gastro-esophageal junction.^[14]^ The inferior intrathoracic portion of the esophagus is the most fragile part of the organ, in that it is relatively poor in longitudinal muscle fibers and is not protected by anatomical structures; moreover, the presence of a large number of vascular and nervous structures weakens the wall.^[15]^ With regard to the anatomical site, complete esophageal perforation occurs in the left lateral wall of the distal esophagus in 90% of cases.^[16]^ In cases of sudden esophageal rupture, the predilection for this site can be explained by several anatomical factors, including the narrowing of the muscle at the level of the distal esophagus, the weakening of this wall owing to the entry of blood vessels and nerves, the lack of support from surrounding structures, and the fact that the esophagus is angled forward at the level of the left diaphragmatic pillar.^[17]^ In the case of Mallory-Weiss syndrome, by contrast, the most common site of the lesion is the right postero-lateral wall, owing to tangential forces, which may cause a tear following a peak in intragastric pressure. Indeed, the posterior and lateral sections of the esophagus are fixed by ligaments and display less elasticity; this means that the probability of laceration of the mucosa in the event of an increase in intragastric pressure is greater in this region than in the left regions (which are more flexible and mobile).^[9]^

Few cases of spontaneous esophageal perforation have been reported in the medical-forensic literature. Clément et al^[18]^ described the case of a 19-year-old subject who died in his sleep as a result of an esophageal rupture which caused mediastinitis, septic shock, and multi-organ failure. Kimura-Katakota et al^[19]^ reported a case of sudden death due to perforation of the inferior third of the esophagus in a 45-year-old man who was asthmatic and alcoholic; the cause of death was ascribed to acute cardio-respiratory failure. In both of these cases, the esophageal tear was found in the left lateral wall of the distal third of the esophagus, which, according to the literature, is the most frequent site of Boerhaave syndrome.

Unlike the above-mentioned cases, in the case reported here we hypothesize that the esophageal rupture was not of sudden onset in a previously healthy esophageal wall; rather, the full-thickness laceration developed from a previous Mallory-Weiss lesion (therefore involving only the mucosa) that had not been diagnosed or treated. This hypothesis stems from the integrated analysis of a set of data gathered from the autopsy, the patient's anamnesis, and the histopathlogical examination.

First of all, the autopsy examination revealed a full-thickness, longitudinal, esophageal tear, measuring about 3 cm in length, in the right lateral wall of the esophagus; as mentioned above, this is statistically the most common site of Mallory-Weiss lesions, while full-thickness ruptures more frequently occur in the left wall. Moreover, intestinal section also revealed the presence of abundant digested hematic material. The sudden rupture of the esophagus does not generally give rise to copious hemorrhage.^[10]^ In addition, the hematic material found both in the intestine and at the death scene was not made up of live red blood that could have resulted from a recent hemorrhage (i.e., at the moment of the rupture). Rather, this abundant material consisted of digested blackish blood (the hemoglobin had undergone a process of degradation) which was indicative of a previous hemorrhage. This finding enabled us to establish that, before the fatal perforation, bleeding had occurred in the upper digestive tract, probably not copiously but intermittently, producing a gradual loss of blood due to a tear in the esophageal mucosa. The bleeding was probably due to a lesion of the mucosa and of the underlying congested venous plexus. Furthermore, the origin of this bleeding could not be explained by the possible coexistence of other pathologies, in that the autopsy and histopathological examination did not reveal the coexistence of any other conditions able to cause digestive hemorrhage (such as esophageal varices, peptic ulcer, erosive gastritis, duodenitis, or angiodysplasia). Secondly, analysis of the anamnesis, as reported by the subject's family members, revealed that the subject, who had a history of alcohol abuse, had been suffering from malaise and thoracic and epigastric pain for about a week without consulting a doctor; only on the day of his death, when the pain symptoms due to the full-thickness esophageal perforation probably worsened, did he call the emergency services, 20 minutes before he was found dead.

On the basis of these findings, it was therefore hypothesized that the subject was suffering from a Mallory-Weiss lesion (probably due to a previous barotrauma, such as a bout of vomiting) which had been causing symptoms (general malaise and thoraco-abdominal pain) for a week. This lesion probably did not elicit massive bleeding, which would have caused the subject's condition to precipitate rapidly; rather, the hemorrhage, which was probably mild and intermittent (as indicated by the presence of digested hematic material in the intestinal loops), had given rise to obscure, aspecific symptoms. On the day of his death, a further barotrauma caused by vomiting (as observed at the death scene) appears to have deepened the pre-existing wall lesion, causing a full-thickness rupture of the esophageal wall, which rapidly led to death.

Toxicological tests carried out on the body fluids proved negative, indicating that the subject had not consumed alcohol shortly before death. Nevertheless, in this case too, a history of alcohol abuse emerged (confirmed by the marked hepatic steatosis observed during autopsy), which could have facilitated the formation of the esophageal tear. The effect of alcohol consumption in the pathogenesis of esophageal lesions lies in the fact that alcohol can, directly or indirectly, cause vomiting. Moreover, alcohol acts on the gastric and esophageal mucosa to increase the retrodiffusion of hydrogen ions, reducing their protective effect; in addition, intoxication by alcohol can alter motor activity and pressure at the level of the inferior esophageal sphincter.^[9]^ Finally, on the days prior to death, any possible alcohol intoxication and its anesthetizing effect could have masked or attenuated the underlying symptoms of an esophageal lesion that had been ongoing for at least a week.

Further confirmation of the presence of a wall lesion prior to the complete laceration emerged from the histopathlogical examination of esophageal samples taken from the site of the tear. Firstly, microscopic examination confirmed the vitality of the lesion, revealing the presence of a neutrophil infiltrate in the esophageal submucosa and aspecific signs of injury and inflammation. Moreover, digested alimentary material was found at the site of the tear, and the presence of accumulations of hemosiderin was detected (by means of Perl stain) in the mucosa and submucosa, presumably indicating bleeding prior to complete perforation. In general, owing to the presence of hydrochloric acid and the remains of food, esophageal perforation may give rise to chemical mediastinitis and hemorrhagic necrosis.^[20]^ As a result of negative intrathoracic pressure, the contents of the stomach and bacteria rapidly enter the pleural cavity, causing a state of sepsis. The septic shock due to mediastinitis leads to multi-organ failure and death. The time-lag between the onset of spontaneous perforation of the esophagus and death has been estimated to be 20 to 24 hours, if medical treatment is not undertaken.^[18]^ In the case reported here, death occurred very rapidly. Indeed, the subject was found dead only 20 minutes after he had called the emergency services to report intense thoracic-abdominal pain and vomiting. It can therefore be hypothesized that no more than an hour had elapsed between the onset of vomiting (which triggered the rupture and the acute symptoms) and death. Furthermore, it is likely that the subject's state of general malaise and the anemia due to persistent slow bleeding contributed to accelerating the fatal evolution of the disease. In this subject, no signs of mediastinitis or multiorgan failure, nor macroscopic or microscopic signs of sepsis, were observed. Thus, on the basis of the autopsy and histopathlogical findings, the cause of death was ascribed to acute cardio-respiratory failure due to pleural effusion following esophageal rupture. Indeed, in agreement with Kimura-Kataoka et al,^[19]^ it may be hypothesized that the time-lag between perforation and death was sufficient to allow the gastric contents to enter the pleural cavities, thereby causing respiratory failure due to pulmonary compression, but not sufficient to cause sepsis and multi-organ failure.

The case reported here is the first in which the integrated study of anamnestic, necroscopic, and autopsy data has revealed the possible transition of a Mallory-Weiss syndrome to a Boerhaave syndrome. This observation supports the concept that distal esophageal tears, rather than constituting a distinct entity, may be part of a spectrum of diseases,^[21]^ and that a partial lesion of the esophageal wall caused by barogenic injury may evolve into a full-thickness rupture following further barotraumas. Although esophageal pathologies are rarely identified on autopsy as the cause of death, in the event of the finding of an esophageal perforation, it is essential for the forensic pathologist to be able to construct a correct picture of the fatal pathology through careful and combined analysis of the autopsy findings and the anamnestic and histopathological data.

## Author contributions

**Conceptualization:** Maria Cuccì.

**Data curation:** Anna Roncallo.

**Investigation:** Giulio Fraternali Orcioni.

**Methodology:** Fiorella Caputo.

**Supervision:** Francesco Ventura.
